# Role of LPAR3, PKC and EGFR in LPA-induced cell migration in oral squamous carcinoma cells

**DOI:** 10.1186/1471-2407-14-432

**Published:** 2014-06-13

**Authors:** Ingvild J Brusevold, Ingun H Tveteraas, Monica Aasrum, John Ødegård, Dagny L Sandnes, Thoralf Christoffersen

**Affiliations:** 1 Department of Pharmacology, Institute of Clinical Medicine, Faculty of Medicine, University of Oslo, and Oslo University Hospital, Blindern, P.O. Box 1057, Oslo N-0316, Norway; 2 Department of Oral biology, Faculty of Dentistry, University of Oslo, Oslo, Norway

**Keywords:** Carcinoma, Epidermal growth factor receptor, Lysophosphatidic acid, LPA receptors, Cell migration, Invasion, Coculture

## Abstract

**Background:**

Oral squamous cell carcinoma is an aggressive neoplasm with serious morbidity and mortality, which typically spreads through local invasive growth. Lysophosphatidic acid (LPA) is involved in a number of biological processes, and may have a role in cancer cell migration and invasiveness. LPA is present in most tissues and can activate cells through six different LPA receptors (LPAR1-6). Although LPA is predominantly promigratory, some of the receptors may have antimigratory effects in certain cells. The signalling mechanisms of LPA are not fully understood, and in oral carcinoma cells the specific receptors and pathways involved in LPA-stimulated migration are unknown.

**Methods:**

The oral carcinoma cell lines E10, SCC-9, and D2 were investigated. Cell migration was studied in a scratch wound assay, and invasion was demonstrated in organotypic three dimensional co-cultures. Protein and mRNA expression of LPA receptors was studied with Western blotting and qRT-PCR. Activation of signalling proteins was examined with Western blotting and isoelectric focusing, and signalling mechanisms were further explored using pharmacological agents and siRNA directed at specific receptors and pathways.

**Results:**

LPA stimulated cell migration in the two oral carcinoma cell lines E10 and SCC-9, but was slightly inhibitory in D2. The receptor expression profile and the effects of specific pharmacological antagonist and agonists indicated that LPA-stimulated cell migration was mediated through LPAR3 in E10 and SCC-9. Furthermore, in both these cell lines, the stimulation by LPA was dependent on PKC activity. However, while LPA induced transactivation of EGFR and the stimulated migration was blocked by EGFR inhibitors in E10 cells, LPA did not induce EGFR transactivation in SCC-9 cells. In D2 cells, LPA induced EGFR transactivation, but this was associated with slowing of a very high inherent migration rate in these cells.

**Conclusion:**

The results demonstrate LPA-stimulated migration in oral carcinoma cells through LPAR3, mediated further by PKC, which acts either in concert with or independently of EGFR transactivation.

## Background

Oral cancers, the majority of which are squamous cell carcinomas, are aggressive neoplasms associated with serious morbidity and considerable mortality [1,2]. A typical feature of these tumours is that they spread largely through progressive local invasive growth [3]. Therefore, much effort is currently directed at understanding the biological mechanisms of the invasive behaviour of oral cancers [4]. Cell migration is controlled by several mechanisms, including complex interactions between the tumour and its stroma [5,6]. Many biologically active substances in the microenvironment, including growth factors, chemokines and various other locally active agents, can induce and regulate cell migration and tumour invasiveness. These substances may be produced by the carcinoma cells or the stromal cells, or both, participating in autocrine or paracrine mechanisms. It is important to understand in detail the role of factors that regulate cell migration and the signalling mechanisms by which they exert their effects in oral cancer. Crucial steps in the invasive process have recently received attention as potential treatment targets, acknowledging the fact that without cell migration, no cell invasion and tumour spread will occur [7].

Several receptors may participate in the control of cell migration. Receptor tyrosine kinases (RTKs), which convey signals from polypeptide growth factors, are of fundamental importance in cell regulation, and if deregulated they may be involved in tumorigenesis [8]. Cellular effects mediated by RTKs typically include stimulated proliferation, enhanced viability, and increased migration [8]. Notable examples of RTKs that can stimulate migration are the epidermal growth factor (EGF) receptor (EGFR ), which is the receptor for the EGF family of growth factors, and Met, which is the receptor for hepatocyte growth factor (HGF, also termed scatter factor, SF) [9-11]. Several signalling pathways may be involved in mediating the stimulation of cell migration and invasion exerted through these receptors. We have previously shown that both EGF and HGF stimulate migration through the phosphoinositide 3-kinase (PI3K)/Akt, the MEK/ERK, and the p38 pathways in oral squamous carcinoma cell lines [12]. Another type of receptors that may play important roles in regulation of cell migration is the large family of G-protein-coupled receptors (GPCRs) [13-15]. Members of this receptor family mediate the effects of numerous factors or other stimuli, including many classical hormones and a variety of locally active substances, such as chemokines, bioactive lipids, and other stromal components. They act via selective interactions with specific heterotrimeric G proteins which specifically couple the receptor activation to one or several downstream pathways [16,17]. Through these mechanisms, the GPCRs transduce signals regulating a variety of cellular processes, including proliferation, viability and migratory activity. Some of these effects depend on interaction between the GPCRs and receptor tyrosine kinases, particularly EGFR [16-21].

Lysophosphatidic acid (LPA) is a glycerophospholipid which is present in all animal tissues and cells and is involved in a large range of physiological functions and pathological conditions and may have a role in cancer [22-24]. LPA is produced mainly by the enzyme autotaxin (lysophospholipase D), and it exerts its functions through the activation of one or more of at least six different receptors. The receptors, named LPAR1-6, all belong to the GPCR family, but are coupled to different downstream signalling pathways and cellular responses [24]. As LPA is abundantly present in saliva, it has a large impact on oral epithelial cells and participates in wound healing, at least in part by inducing epithelial cell migration [25]. In oral squamous cell carcinoma (OSCC) cell lines, LPA has been reported to induce migration [26,27]. Due to its ability to induce cell migration and invasion, LPA, its receptors, and autotaxin have been proposed as novel targets for cancer treatment [22,28]. However, LPA has also been found to inhibit migration in melanoma cells, and thereby act as a tumour suppressor [29]. To date, very little information exists about which LPA receptors are present and active in oral carcinoma cell lines.

The aim of this study was to investigate to what extent LPA affects migration in oral cancer cell lines and to examine some of the underlying mechanisms. The work focused particularly on two aspects. First, since little is known about which mechanisms LPA acts through in control of migration, we have begun studies to identify the specific receptors involved. Second, since it has previously been found that other GPCR activators may exert mitogenic effects both by interacting with EGFR signalling and by other mechanisms in various cancer cells [20,21,30], we have investigated the relative roles of EGFR-dependent and EGFR-independent signalling in the regulation of migration in the oral cancer cells.

## Methods

### Materials

LPA (L-α-Lysophosphatidic acid, oleoyl, sodium, cat. # L7260), neurotensin (NT), 12-O tetradecanoylphorbol-13-acetate (TPA), epidermal growth factor (EGF) and GF109203X hydrochloride were obtained from Sigma-Aldrich, (Saint Louis, MO, USA). CXCL12 (recombinant human CXCL12/SDF-1γ) was from R&D Systems (Minneapolis, MN, USA). Dodecylphosphate (LP-105) was from Enzo Life Sciences (Farmingdale, NY, USA). 1-oleoyl-2-methyl-*sn*-glycero-3-phosphotionate ((2S)-OMPT) and N-{(1R)-2-hydroxy-1-[(phosphonooxy)methyl]ethyl}(9Z)octadec-9-enamide (VPC31143(R)) were from Avanti Polar Lipids (Alabaster, AL, USA). GM6001 (CAS 142880-36-2) was from Calbiochem (Merck KGaA, Darmstadt, Germany). Prostaglandin E2 (PGE2) was from Cayman (Ann Arbor, MI, USA). Gefitinib and cetuximab were kind gifts from AstraZeneca (Division Oslo, Norway) and Merck-Serono (Merck KGaA, Darmstadt, Germany), respectively. Ki16425 was from Santa Cruz Biotechnology (CA, USA). Anti-phospho-EGFR (Tyr 1173) was from Invitrogen (Paisley, UK), anti-ERK1/2 (cat. # 4695), anti-p-ERK1/2 (Thr202/Tyr204, cat. # 9106), anti-p-p38 (Thr180/Tyr182), anti-phospho-Akt (Ser 473) and anti-GAPDH was from Cell Signaling (Danvers, MA, USA) and anti-LPAR1/2/3 were from LSBio (LifeSpan BioSciences Inc., WA, USA). Secondary goat anti-mouse and goat anti-rabbit IgG HRP-conjugated antibodies were purchased from Bio-Rad Laboratories (Hercules, CA, USA). The inhibitors SB203580, PD98059 and LY294002 were all purchased from Calbiochem (Merck KGaA, Darmstadt, Germany) and dissolved in DMSO (Sigma-Aldrich). Smart pool human On-target plus LPAR3 siRNA and Non-targeting Control Pool were from Thermoscientific (GE Healthcare, Dharmacon Inc.).

### Cell culture

PE/CA-PJ-49 clone E10 (hereafter termed E10, ECACC, Salisbury, UK, originally supplied by Drs Kosmehl and Berndt, Friedrich-Schiller University, Jena, Germany) were from a tongue squamous cell carcinoma in a 57-year old male patient. PE/CA-PJ41 (clone D2) (hereafter termed D2, ECACC, originally supplied by Drs Kosmehl and Berndt, Friedrich-Schiller University, Jena, Germany ) were from the oral squamous epithelium of a 67 year old female. SCC-9 (ATCC, Manassas, VA, USA) were from a tongue squamous cell carcinoma in a 25-year old male patient. The E10 and D2 cells were cultured in Iscove’s modified Dulbecco’s medium (IMDM)(Sigma-Aldrich, St. Louis, MO,USA) supplemented with 10% FBS (Lonza, Walkersville, MD, USA), 2 mM L-glutamine (Cambrex), 1% Pen/Strep (Gibco). The SCC-9 cells were cultured in DMEM-Ham’s F12 medium (cat. # 12-615 F, Lonza) supplied with 400 ng/ml hydrocortisone, 1% Pen/Strep (Gibco) and 10% FBS. All cell lines were subcultured by trypsination (Trypsin-EDTA, Lonza). Normal human fibroblasts were obtained from healthy adults with written consent, as described previously [31].

**Genotyping** by the Powerplex® 16 system (Promega) was performed at the Norwegian Radium Hospital, Department of Tumour Biology, Oslo, Norway.

### Wound scratch assay

Cell migration was monitored in a wound scratch assay as described previously [12]. Briefly, a scratch was made with a sterile 100 μl pipette tip in a confluent cell layer, washed twice in physiologic saline, and then various stimulatory or blocking agents were added in serum-free medium. Wells were photographed at the beginning of the experiment and after 24 h (E10-cells), 17 h (D2-cells) or 24 and 48 h (SCC-9 cells). Pictures were obtained with an F-view camera and AnalySIS Image processing software mounted on an Olympus IX81 inverted microscope with a 4x objective (Olympus Norge AS, Oslo, Norway). AxioVision Rel.4.8 software (Carl Zeiss, Oslo, Norway) was used for analysis.

### Organotypic 3D cocultures

Three-dimensional coculture models were prepared as described previously [31]. Briefly, normal human fibroblasts from oral mucosa were embedded in a collagen matrix. E10 cells were seeded on top of the matrix the next day. At day 5, the cultures were lifted to air-liquid interface, resting on the membrane of a transwell insert with the medium below the membrane. LPA was added to the medium at every medium exchange from day 4 and throughout the protocol until harvesting the tissue at day 11. The organotypic tissue was fixed in 4% paraformaldehyde, embedded in paraffin and cut into 4 μm sections, mounted on glass slides and stained with hematoxylin and eosin.

### Western blotting

50.000 cells were seeded in each well of 12-well plates. The cells were grown for 24 hours in complete IMDM (Sigma-Aldrich), then in serum-free medium for 24 hours before stimulation with LPA with or without specific inhibitors as indicated in Results. After stimulation, cells were washed once with PBS, and scraped directly in Laemmli buffer (4% SDS, 20% Glycerol, 120 mM Tris–HCl, pH6.8, 0.006% bromphenol blue and 10% mercaptoethanol), and aliquots of 20 μg protein were separated on 10% polyacrylamide gels by electrophoresis under denaturing conditions. The proteins were transferred to nitrocellulose membranes using a semidry transfer system (Bio-Rad). The membranes were incubated with primary antibody in Tris-buffered saline containing 0.1% Tween 20 (TBST) with 5% non-fat dry milk or BSA overnight at 4°C. The blots were then washed three times in TBST and incubated with HRP-conjugated secondary antibodies at room temperature for 1 h. The blots were visualized with LumiGLO® (KPL, Gaithersburg, MD).

### siRNA transfection

E10 cells seeded in 12-well plates were transfected with siRNA smart pool targeting human LPAR3 mRNA 3 h after plating by the use of Lipofectamine 2000. The medium was replaced and 100 μl transfection mixture containing 3 μl Lipofectamine 2000 (Invitrogen, Carlsbad, Ca) and 3 μl 20 μM LPAR3 ON-TARGET plus siRNA (Dharmacon, Lafayette, CO) in OptiMEM was added pr well (1 ml medium), giving a final concentration of 60 nM siRNA. Control cells were transfected with the same amount of ON-TARGET plus Non-targeting siRNA (Dharmacon). Transfected cells were then cultured for 72 h and harvested for qPCR. The same procedure was applied to cells seeded for the wound scratch assay, where the wound was made 72 h after transfection.

### NanoPro isoelectric focusing

50.000 cells were seeded in each well of 12-well plates. The cells were grown for 24 hours in complete IMDM (Sigma-Aldrich), then in serum-free medium for 24 hours before stimulation with LPA with or without specific inhibitors as indicated in Results. Cells were lysed with Bicine/CHAPS buffer with aqueous and DMSO inhibitor mixes (Protein Simple, Santa Clara, CA, USA) on ice. Lysates (0.1 mg/ml) were mixed with fluorescent pI Standard Ladder 3, Ampholyte premix G2, pH 5–8 separation gradient (Protein Simple) and loaded into capillaries in a Cell Biosensis Protein Simple NanoPro 1000 system according to the manufacturer’s instructions. Proteins were separated by capillary isoelectric focusing separation performed at 21 mW for 40 minutes and then immobilized with UV light exposure for 70 s. Anti-ERK1/2 antibody or anti-phospho ERK1/2 was then applied to the capillaries and probed with secondary anti-mouse IgG. The NanoPro machine performed automated wash steps with Wash buffer (Protein Simple). Primary antibody was incubated for 2 hours and secondary antibody for 1 hour. Signal was detected with Luminol and Peroxide (Protein Simple) and imaged with a CCD camera. Quantitation was performed with Compass software (Protein Simple). The method is described and validated by O’Neill et al. [32].

### RT-PCR

*Isolation of mRNA*: RNAwas isolated with QIAGEN RNeasy kit (Cat.No. 74106) according to the manufacturer’s description. RNA was treated with DNAse (RNase-Free DNase Set, QIAGEN).

*cDNA synthesis*: cDNA was synthesized from 2.5 or 5 μg RNA with Superscript® III reverse transcriptase (Invitrogen, CA, USA) with oligo(dT) as primer according to the manufacturer’s recommendation. Primers: Primer sequences were designed by use of NCBI/Primer-BLAST software [33]. Primers used in this study with NCBI reference sequences (Invitrogen, CA): *LPAR1*: Forward primer AAGCTCCCCATCCACCTATCT, Reverse primer CATTCATGGCTGTGAACTGGG. *LPAR2*: Forward primer CTTCTACGTGCGGCGGCGAG, Reverse primer ACCACGAACGCCCCCAGGA. *LPAR3*: Forward primer CGGGTGAACGTGAGCGGATGT, Reverse primer TCACTGCCGCGATGACCAGA. *LPAR4*: Forward primer GCGGTTTGCAGTAAAAAGCTGCGG, Reverse primer TTTCCTCCCCAAGAAAGAGTGTGCT. *LPAR5*: Forward primer CCCTGAGGAGGTCTCTGCTGC, Reverse primer CATGGCATTCACCTCCGGGGC. *LPAR6*: Forward primer TCCCTCTGCTATGGCTCTTCCTCA, Reverse primer TGAGGCCTTTTCCTCAGTTGCCA.

*Real-time quantitative PCR (qRT-PCR)*: PCR assays were analysed with an Applied Biosystems 7900HT Fast Real-Time PCR System using Platinum SYBR Green qPCR Supermix-UDG with ROX (cat. # 11744, Invitrogen, CA). Data were analysed with the SDS software (ver. 2.2, Applied Biosystems), cycle of threshold (Ct) and variation in baseline were calculated from each amplification plot. Based on the Ct value and standard curves the relative input amount of mRNA was calculated. The data were normalized using GADPH as internal control.

### Statistical analysis

Statistical analysis for the migration studies was performed using Sigmaplot 11.2 (Systat software, Inc., San Jose, CA, USA). Mean percent wound closure of groups was compared using t-test for normally distributed data and Mann–Whitney rank-sum test when data were not normally distributed. A difference was considered to be statistically significant where the corresponding p-value was ≤0.05. Exact p-values are given in figure legends.

Densitrometic analyses of immunoblots were obtained with ImageJ (National Institutes of Health, Bethesda, Maryland, USA). Data were expressed as mean ± SEM from independent experiments and visualized with GraphPad Software. The statistical significance of differences was analysed by unpaired t-test using GraphPad Software.

## Results

### Effect of LPA on migration and invasive activities in OSCC cell lines

**Figure 1:**
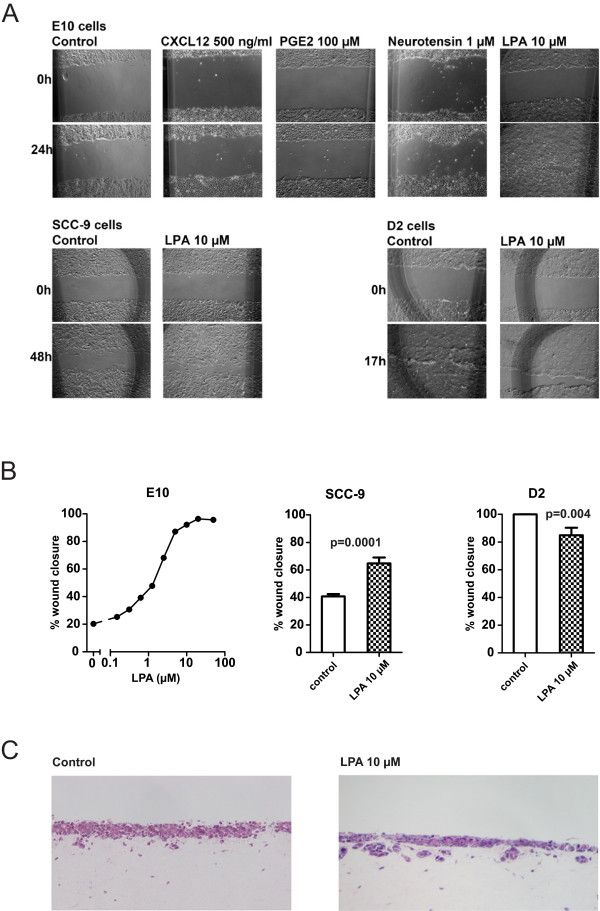
**Effect of LPA on migration and invasiveness in oral carcinoma cells. A**: *The effect of various GPCR ligands on cell migration.* Cell migration was observed after scratch wounding in a confluent cell layer. Various GPCR ligands were added to the wells with serum-free medium as indicated. Digital images were obtained immediately after stimulation and after 17 h (D2), 24 h (E10) or 48 h (SCC-9) and wound closure was measured. The dark marks on the images were made to orient the picture and ensure that they were from the same location. **B**: *Dose-dependent wound closure with LPA stimulation in the E10, SCC-9 and D2 cells.* Cell migration was measured after scratch wounding in a confluent cell layer as in A. LPA was added to the wells with serum-free medium as indicated. Digital images were obtained immediately after stimulation and after 17 h (D2), 24 h (E10) or 48 h (SCC-9) and per cent wound closure was measured. Bars represent mean ± SEM (Standard error of the mean), n = 5. Left: Dose/response curve for one experiment in E10 cells is shown with doses from 0 to 100 μM, indicating a near maximal effect at 24 h using 10 μM LPA. 10 μM of LPA was chosen for use in further experiments. **C**: Organotypic coculture model without (left) and with (right) 10 μM LPA added during day 4 to 11 of culture.

We first tested the effects of several GPCR agonists on migration, using scratch wound healing in the E10 cell line as an experimental model. In previous work on these cells we determined optimal conditions for such studies, demonstrated strong effects of both EGF and HGF on the migration, and could investigate underlying signalling mechanisms [12]. While neither CXCL12, PGE_2_ nor NT were found to be powerful activators of migration in E10 cells, LPA induced a strong dose-dependent migratory response, which at 24 h was maximal at 10 μM, with ED50 at about 2 μM (Figure 1A,B). Furthermore, we examined the effect of LPA on two additional oral carcinoma cell lines. We found that LPA also stimulated cell migration in the SCC-9 cells (Figure 1A,B). On the other hand, LPA had a slightly inhibitory effect on migration in the D2 cells, a cell line that is highly migratory without stimulation. We observed full wound closure in the D2 cells by approximately 17 h in serum-free medium without LPA, while the migration was slower and wound closure occurred several hours later in the presence of 10 μM LPA. In further experiments, 10 μM LPA was chosen as the preferred dose for all the cells, and readout for cell migration was set at 24 h for the E10 cells, 48 h for the SCC-9 cells, and 17 h for the D2 cells.

To examine if stimulation of migration by LPA was reflected in enhanced invasiveness, we tested the capacity of LPA to induce cellular invasion in three-dimensional (3D) culture. For this purpose we used an organotypic 3D model consisting of human oral fibroblasts embedded in a collagen I matrix with E10 carcinoma cells seeded on top [31]. After 11 days of co-culture, a multilayer squamous carcinoma epithelium had formed on top of the matrix (Figure 1C). The 3D cultures were kept with or without LPA in the medium. We consistently found that LPA increased the tendency of the carcinoma cells to invade the fibroblast/collagen layer, with more tumour cell islands in the connective tissue compartment than in untreated controls (Figure 1C).

### Expression of LPA receptors in OSCC cell lines

**Figure 2:**
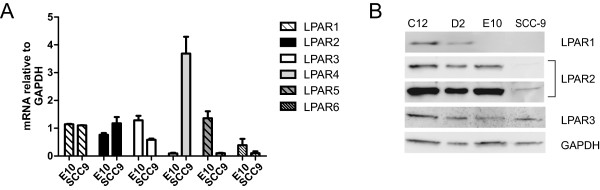
**LPA receptors present in oral carcinoma cell lines.** mRNA and protein levels were detected in lysates from subconfluent, unstimulated cells. **A**: qRT-PCR shows that mRNA encoding LPAR1-6 was present in unstimulated E10 and SCC-9 cells. n = 4. Bars represent mean ± SEM. **B**: Western blots show LPAR1 (at 41 kDa), LPAR2 (at 39 kDa) and LPAR3 (at 40 kDa) protein in unstimulated E10 and SCC-9 cells. n = 3. Due to a very weak LPAR2 band in the SCC-9 cells, the same blot is shown twice with normal and increased exposure for visualisation.

We next started studies aiming at understanding which receptors are mediating the effects of LPA on migration in the oral carcinoma cells. Present evidence indicates that there are at least six different LPA receptors [34]. Studies in other cells have shown varying expression of LPA receptors. Qualitative RT-PCR revealed that both the E10 and the SCC-9 cells expressed LPAR1, 2, 3, 4, 5, and 6 mRNA at different levels (Figure 2A). For protein expression, we focused on the EDG-family members of LPA receptors, i.e. LPAR1-3. Antibodies against LPAR4-6 did not show adequate specificity in our cells and were not used. The LPAR1 protein was not expressed on Western blots in the E10 and the SCC-9 cells, but was present in the D2 cells. Another oral carcinoma cell line, C12, which was employed for comparison, also strongly expressed LPAR1. All the cells studied expressed LPAR2 and LPAR3 proteins (Figure 2B).

### Effects of pharmacological agents acting on specific LPA receptors

**Figure 3:**
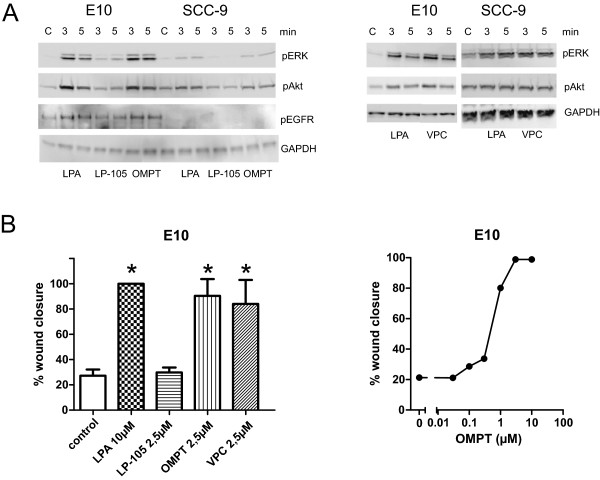
**Effects of LPAR specific agonists.** LP-105 (agonist for LPAR2), VPC31143(R) (agonist LPAR) and (2S)-OMPT (agonist for LPAR3) were used. **A**: Serum-starved E10 and SCC-9 cells were stimulated for 3 or 5 min with LPA, LP-105, OMPT or VPC, and blots were detected for pERK, pAkt and pEGFR. Similar phosphorylation levels were seen with LPA, VPC and OMPT in both cell lines. No phosphorylation of EGFR was detected in SCC-9 cells with any of the agonists. **B**: Cell migration measured in wound assays. Left panel: E10 cells were stimulated with LPAR-agonists as indicated and migration was measured at 48 h, n = 3. Bars represent mean ± SEM. *indicates p < 0.05 compared to control. VPC (2.5 μM) and OMPT (2.5 μM) gave similar results as LPA (p < 0.05), while no migration was induced by LP-105. Right panel: Dose–response curve for LPAR3 agonist OMPT in E10 cells from one representative experiment show full wound closure with 2.5 μM OMPT at 48 h.

**Figure 4:**
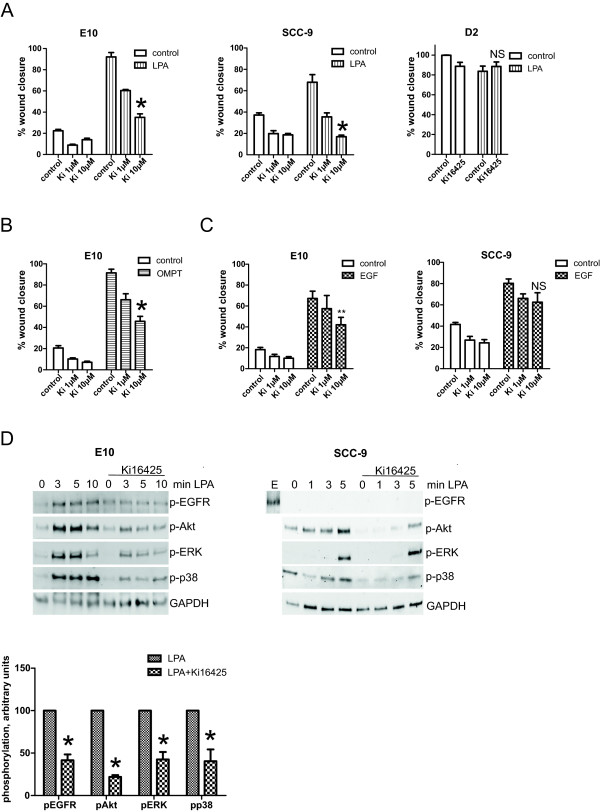
**Inhibition of LPAR1 and 3 with Ki16425. A-C**: *Effect on cell migration.* Cell migration was measured after scratch wounding in a confluent cell layer. A: Inhibitor, solvent (DMSO) and/or LPA were added to the wells with serum-free medium as indicated. Digital images were obtained immediately after stimulation and after 24 h (E10), 48 h (SCC-9) and 17 h (D2) and wound closure was measured. **B**: Inhibitor, solvent (DMSO) and/or LPAR3 agonist OMPT at 48 h gave similar results as LPA at 24 h in E10 cells. **C**: Inhibitor, solvent and/or EGF were added as indicated in E10 and SCC-9 cells. Digital images were obtained immediately after stimulation and after 24 h (E10), 48 h (SCC-9). Bars represent mean ± SEM, n = 3 or 4. The following concentrations were used: LPA 10 μM, OMPT 2.5 μM, EGF 5 nM, Ki16425 1 or 10 μM as indicated. * indicates p < 0.001, **indicates p = 0.03, NS = Not significant. **D**: *Effects on EGFR, Akt, ERK and p38 phosphorylation.* Subconfluent cell cultures were pre-treated with Ki16425 for 30 min, and then stimulated with 10 μM LPA for 3, 5, 10 min (E10 cells) or 1, 3, 5 min (SCC-9 cells) as indicated. Cells were lysed for Western blotting. Western blots show partial inhibition of Akt, ERK, p38 and EGFR phosphorylation in E10 cells (D left), while in SCC-9 cells (D right) very little inhibition was detected. No EGFR phosphorylation with LPA was observed in SCC-9 cells. As a positive control, EGFR phosphorylation with EGF stimulation (abbreviated E) is shown. Densitometric analyses of E10 cell at 5 min stimulation are shown below the E10 blots. *indicates p ≤ 0.01.

To further study the role of the different LPA receptors, we examined functional response in cells treated with different receptor-specific agonists, focusing on LPAR1-3 (Figure 3). First, we used the agonist VPC31143(R), originally thought to be specific for LPAR1 [35]. This agonist stimulated phosphorylation of ERK in E10 and SCC-9 cells (Figure 3A). However, more recently it has been shown that VPC31143(R), a NAEPA (N-acyl ethanolamide phosphate)-derived LPA agonist, activates all the LPARs [34], which is more compatible with the expression data since LPAR1 protein was not detected in E10 or SCC-9 cells (Figure 2). To our knowledge, other more specific LPAR1 agonists are not available at the moment. The LPAR2-specific agonist LP-105 (also named FAP12) gave only a weak phosphorylation of EGFR, Akt, and ERK as compared to LPA in the E10 cells, and no detectable phosphorylation of EGFR, Akt, or ERK in the SCC-9 cells (Figure 3A). However, the LPAR3-specific agonist (2S)-OMPT readily induced phosphorylation of EGFR, Akt, and ERK in the E10 cells and, more weakly, in SCC-9 (Figure 3A). Using E10 cells as a model, we also found that (2S)-OMPT induced cell migration of about the same magnitude and slightly higher potency than LPA, with a maximal effect at ≥2.5 μM and ED50 at about 0.5 μM (Figure 3B, right). VPC31143(R) also stimulated, while LP-105 had no effect on migration in the E10 cells (Figure 3B).Very few commercially available LPAR inhibitors exist, and they mainly target LPAR1 and/or LPAR3. The LPA inhibitor Ki16425 is known to inhibit both LPAR1 and LPAR3. We found that Ki16425 inhibited the ability of LPA to induce migration in both the E10 and SCC-9 cells, which, in view of our failure to demonstrate expression of LPAR1 protein, is additional support for LPAR3 being involved (Figure 4A). However, in the E10 cells, the inhibition was not complete, suggesting that these cells might have other active LPA receptors. Ki16425 also inhibited the migration induced by the LPAR3-specific agonist (2S)-OMPT in E10 cells, providing further support for LPAR3 as a mediator of the LPA effect (Figure 4B). In the SCC-9 cells, the Ki16425 completely inhibited the LPA-induced migration, decreasing it to a level below the controls, suggesting a basal activity of LPAR3 in the SCC-9 cells (Figure 4A). In the D2 cells, no significant effect of Ki16425 on migration was observed (Figure 4A). Ki16425 also had a partial inhibitory, statistically significant effect on EGF-induced cell migration in E10 cells (p = 0.03), while the effect in SCC-9 cells was not significant (Figure 4C). We then investigated the effect of the LPAR blocker Ki16425 on LPA-induced phosphorylation of signalling molecules. In the E10 cells, Ki16425 inhibited, although not completely, the phosphorylation of EGFR, Akt, ERK and p38 (Figure 4D). Although LPA-induced migration was inhibited with Ki16425 in the SCC-9 cells, this inhibitor had no effect on the immediate phosphorylation of ERK, but slightly reduced Akt and p38 phosphorylation (Figure 4D).

**Figure 5:**
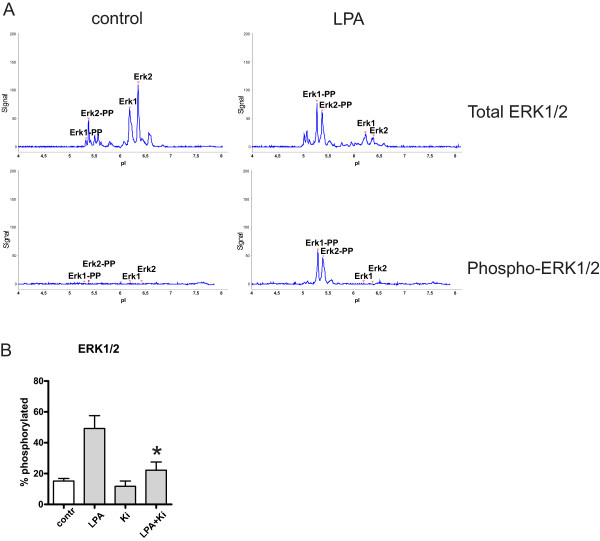
**Effects of LPA and Ki16425 on ERK1/2 phosphorylation assessed by isoelectric focusing with NanoPro 1000.** Subconfluent cell cultures of E10 cells were untreated or pre-treated with Ki16425 for 30 min, and then stimulated with 10 μM LPA and lysed for isoelectric focusing with NanoPro detection system as described in Methods. **A**: Typical pI spectra. Total ERK was detected in control (upper left) and LPA-stimulated E10 cells (upper right). Peaks were verified with a phospho-ERK antibody in control (lower left) and LPA-stimulated cells (lower right). **B**: ERK phosphorylation in E10 cells in response to LPA with or without Ki16425 before LPA stimulation. n = 3. Bars represent mean ± SEM. *indicates p = 0.018.

**Figure 6:**
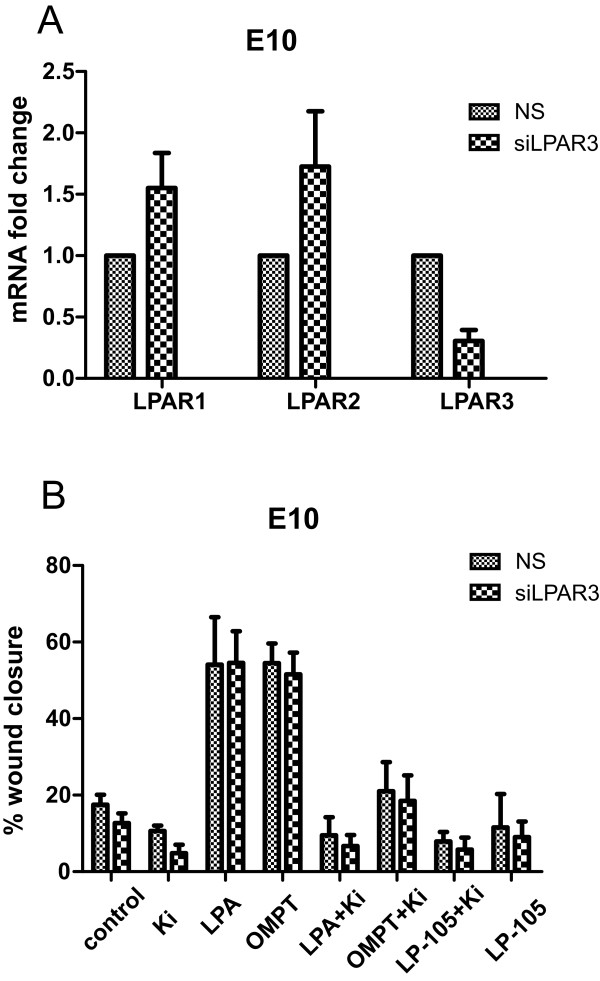
**Effects of siRNA down regulation of LPAR3. A**:*Downregulation of LPAR3 with siRNA leads to a concomitant upregulation of LPAR1 and 2. *Cell cultures were treated with 60 nM siLPAR3 or non-silencing pool with transfection reagent (NS) for 72 h before harvest. qRT-PCR data demonstrate a downregulation of LPAR3 compared to NS. LPAR1 and LPAR2 were upregulated at the same time. **B**: *Cell migration in siLPAR3-treated cells.* Cells seeded to confluence as in the other migration experiments. Transfecction reagents were added right after seeding, and cells were incubated for 72 h before the scratch was made. The cells were then treated with LPA and/ or Ki or LPAR2-agonist LP-105 the same ways as described for the other experiments.

To further validate the results obtained with LPA and inhibitors, we assessed some of the responses with isoelectric focusing as a supplement to Western blots. ERK1/2 phosphorylation in E10 cells was used as a model, and isoelectric focusing combined with immunodetection was performed with the NanoPro system [32]. Figure 5A shows a typical pI spectrum for ERK1/2 probed with antibody against total ERK1/2 in unstimulated (upper left) and LPA-stimulated (upper right) cells. The profile shows that upon LPA treatment, there was a shift from unphosphorylated to phosphorylated ERK1/2 signals. The peaks corresponding to phosphorylated ERK were verified with a phosphospecific ERK antibody (lower panels). The low level of phosphorylated ERK seen in the unstimulated samples with the pan-ERK antibody, as also seen in the Western blots, was not detected in the NanoPro system with the phosphospecific antibody, for reasons that we at present cannot fully explain. Figure 5B shows quantification of data, based on the use of the pan-ERK antibody, from 3 experiments generated in principle as in Figure 5A. The results demonstrate that the stimulation of ERK phosphorylation after addition of LPA was significantly inhibited by Ki16452 (p = 0.018). These data are well in accordance with the findings with immunoblotting (Figure 4D). Using the NanoPro technique, we have also performed similar analyses of the effects of other blockers of specific pathways (see below).To further examine the effect of LPAR3 on LPA-stimulated cell migration, we pretreated the cells with siRNA against LPAR3 (Figure 6). The LPAR3 siRNA did not block LPA-induced cell migration, but the migration was still inhibited by the use of LRAR1/3-inhibitor Ki16425 (Figure 6B). We then examined the expression of LPAR1 and LPAR2 upon the use of LPAR3 siRNA, and found that LPAR1 and 2 mRNA were upregulated while LPAR3 mRNA was (partly) downregulated (Figure 6A). This suggests that LPAR3 was not sufficiently suppressed and/or that LPAR1 may take over as an inducer of cell migration when LPAR3 has been downregulated. The effect of LPAR2 was examined using the specific agonist LP-105. Although we saw an upregulation of LPAR2 mRNA after LPAR3 silencing, this agonist did not induce migration, neither in the absence nor the presence of the concomitant use of LPAR1/3 inhibitor.

### Role of protein kinase C (PKC), downstream pathways and EGFR in LPA-induced migration

**Figure 7:**
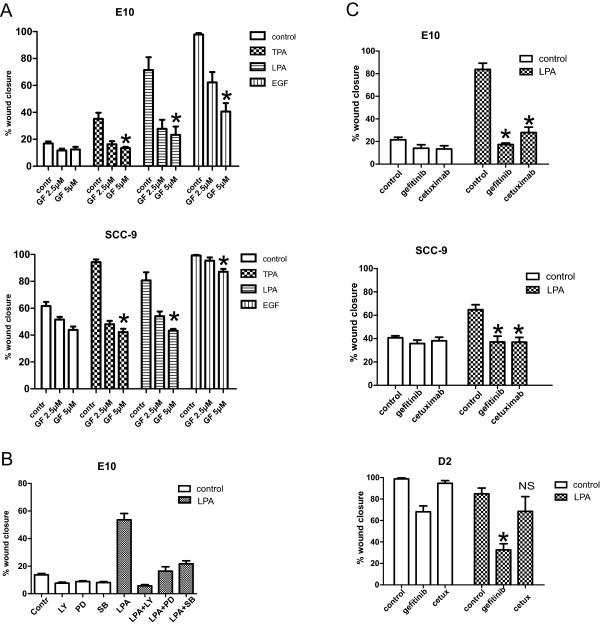
**Role of PKC and EGFR in LPA-induced cell migration. A**: *Role of PKC:* Cell migration after scratch wounding in a confluent cell layer. Inhibitor, solvent (DMSO) and/or TPA (0.01 μM), LPA (10 μM) or EGF (5 nM) was added to the wells with serum-free medium as indicated. Digital images were obtained immediately after stimulation and after 24 h (E10) or 48 h (SCC-9). PKC inhibitor (GF109203X) abolished LPA-induced cell migration slightly dose-dependent (stronger inhibition with 5 μM than with 2.5 μM). TPA gave a small induction of cell migration in the E10 cells and a larger induction in the SCC-9 cells that was inhibited by GF, confirming PKC-involvement. n = 3. **B**: Role of downstream signalling pathways: Cell migration after scratch wounding in a confluent cell layer: Inhibitor, solvent (DMSO) and/or LPA were added to the wells with serum-free medium as indicated. Digital images were obtained immediately after stimulation and after 24 h. **C**: *Role of EGFR:* Cell migration after scratch wounding in a confluent cell layer. Inhibitors, solvent (DMSO) and/or LPA were added to the wells with serum-free medium as indicated. Digital images were obtained immediately after stimulation and after 24 h (E10), 48 h (SCC-9), and 17 h (D2) and wound closure was measured. Bars represent mean ± SEM, n = 3, 4 or 5. LPA 10 μM, gefitinib 1 μM, cetuximab 0.16 μM, GF109203X 2.5 μM and 5 μM, LY294002 10 μM, PD98059 10 μM, SB203580 10 μM. *indicates p < 0.001.

We studied the possible role of protein kinase C (PKC) in regulation of LPA-induced migration. PKC has been found to play a role in many cancer cells [36]. We examined whether the LPA-stimulated migration was affected by inhibition of PKC. In both the E10 and the SCC-9 cells, treatment with the PKC inhibitor GF109203X almost completely inhibited LPA-induced cell migration (Figure 7A). Further evidence of a role for PKC was obtained with the use of tetradecanoyl phorbol acetate (TPA), a direct activator of PKC. TPA induced cell migration in both E10 and SCC-9 and this effect was inhibited by GF109203X (Figure 7A). Addition of the PKC inhibitor before the stimulation with EGF resulted in a considerable inhibition of the migration in the E10 cells, but only had a minor, however significant inhibitory effect in the SCC-9 cells (Figure 7A).The importance of downstream kinase pathways in cell migration was studied. Blocking of the MEK/ERK kinase, the p38 kinase and the PI3 kinase with PD98059, SB203580, and LY294002 respectively, all inhibited the LPA-induced cell migration in the E10 cells (Figure 7B).

EGF was previously found to strongly stimulate migration in several oral cancer cells, including E10 [12], and transactivation of EGFR has been found to be part of the mechanism of mitogenic effects of GPCRs in several cancer cells [20,21,30,37]. We now investigated the role of EGFR signalling in the stimulation of migration by LPA. Blocking of the EGFR with the EGFR tyrosine kinase inhibitor gefitinib or the EGFR antibody cetuximab inhibited LPA-stimulated cell migration down to control level or below in E10 and SCC-9 cells (Figure 7C, top and middle panel). This suggests a role for EGFR in the cellular response to LPA, in terms of either a necessary synergism between downstream signalling pathways or an LPA-induced transactivation of EGFR. In the D2 cells gefitinib, but not cetuximab, reduced the inherent cell migration, conceivably reflecting differences related to inhibition of the extracellular ligand binding site versus the intracellular kinase site. The effect of gefitinib in D2 cells was more pronounced in the presence of LPA (Figure 7C, bottom panel).

### EGFR and downstream pathways in LPA signal transduction

**Figure 8:**
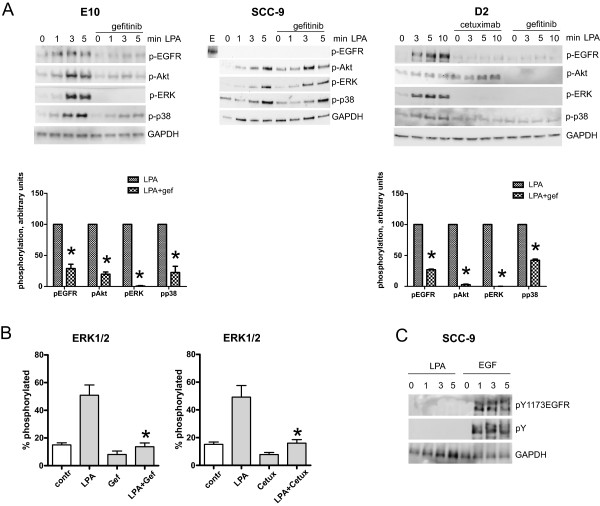
**Role of EGFR in LPA signal transduction.** Subconfluent cell cultures were pre-treated with 1 μM gefitinib for 30 min before stimulation with 10 μM LPA for 1, 3 or 5 min as indicated. Cells were lysed for Western blotting or isoelectric focusing with NanoPro detection system as described in Methods. **A**: Western blots show phosphorylation of EGFR, Akt, ERK and p38 after LPA stimulation. The phosphorylation was inhibited by gefitinib pre-treatment in E10 and D2 cells. Cetuximab, in contrast to gefitinib did not inhibit Akt phosphorylation in D2 cells. In SCC-9 cells, Akt, p38, and ERK were phosphorylated by LPA, but not inhibited by gefitinib. No phosphorylation of EGFR was detected after LPA stimulation in the SCC-9 cells. As a positive control, EGFR phosphorylation after EGF stimulation was shown. n = 3. Graphs showing blot quantifications for E10 and D2 at 5 min are shown below the blots. **B**: NanoPro detection confirmed inhibition of LPA-induced ERK phosphorylation with gefitinib (1 μM) in E10 cells. Similar results were seen with cetuximab (0.16 μM). **C**: Western blots show tyrosine phosphorylation of EGFR and total tyrosine phosphorylation in SCC-9 cells after stimulation with EGF. No tyrosine phosphorylation is detected after LPA stimulation. n = 3 *indicates p ≤ 0.01.

Figure 8A shows that in E10 cells stimulated with LPA, there was a marked and rapid phosphorylation of EGFR (at Tyr 1173) as well as the downstream signalling molecules ERK, Akt, and p38. When E10 cells were treated with gefitinib, the LPA-induced phosphorylation of EGFR was blocked, the effects on Akt and p38 were strongly inhibited, and the effect on ERK was completely abolished. The effect of gefitinib and cetuximab on ERK phosphorylation in LPA-stimulated E10 cells was studied further by use of isoelectric focusing in the NanoPro detection system. Here, we could demonstrate that LPA induced phosphorylation of ERK1/2, and that the phosphorylation was inhibited when the cells were pre-treated with cetuximab or gefitinib, confirming the Western blot results (Figure 8B). In contrast to the E10 cells, SCC-9 cells treated with LPA did not exhibit any phosphorylation of EGFR or any other tyrosine phosphorylation corresponding to the size of the ErbB family protein detected with a phosphotyrosine antibody (Figure 8A, middle and 8C). For comparison, EGF induced strong tyrosine phosphorylation in these cells (Figure 8C). In these cells LPA induced strong phosphorylation of ERK, Akt, and p38, but these effects were not sensitive to gefitinib. The D2 cells responded to LPA in a manner very similar to the E10 cells, as EGFR, ERK, and Akt were phosphorylated, and these effects were inhibited by gefitinib (Figure 8A, right). Interestingly, cetuximab did not inhibit Akt in the D2 cells, which may reflect properties of the EGFR system in these cells and corresponds to the failure of cetuximab to inhibit migration in D2 cells.

**Figure 9:**
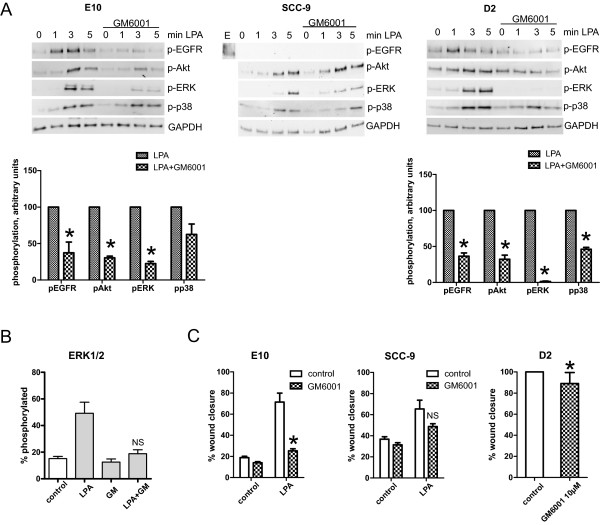
**Role of release of membrane-bound ligand in EGFR transactivation. ***Involvement of membrane-bound EGFR ligand in LPA signal transduction was examined by use of MMP-inhibitor GM6001.* Subconfluent cell cultures were pre-treated with 10 μM GM6001 for 30 min, then stimulated with 10 μM LPA for 1, 3 or 5 min as indicated. Cells were lysed for Western blotting or isoelectric focusing with NanoPro detection system as described in Methods. **A**: Western blots show the inhibition of LPA-induced EGFR, Akt and ERK phosphorylation in E10 and D2 cells, but no inhibition in SCC-9 cells. Graphs showing blot quantifications for E10 and D2 at 5 min are shown below the blots. **B**: NanoPro detection confirmed the partial ERK inhibition in E10 cells, however not statistically signinficant. **C**: *Role of membrane-bound ligand in cell migration.* Cell migration was detected after scratch wounding in a confluent cell layer. Inhibitor, solvent (DMSO) and/or LPA was added to the wells with serum-free medium as indicated. Digital images were obtained immediately after stimulation and after 17 h (D2), 24 h (E10) and 48 h (SCC-9) and wound closure was measured. Bars represent mean ± SEM, n = 3 or 4. LPA 10 μM, GM6001 10 μM. *indicates p ≤ 0.05, NS = Not significant.

Endogenously produced EGFR ligands can participate in autocrine mechanisms and mediate EGFR transactivation, thus enhancing EGFR-driven tumorigenesis. LPA has been shown to activate MMP-2 in ovarian cancer [38]. To test if LPA induced release of EGFR ligands in the oral carcinoma cells, we treated them with the matrix metalloprotease (MMP) inhibitor GM6001 prior to stimulation with LPA (Figure 9). In the E10 cells, GM6001 strongly reduced the phosphorylation of EGFR, Akt and ERK, suggesting that LPA transactivated EGFR via the release of endogenously produced EGFR ligands (Figure 9A). The p38 phosphorylation was unaffected. Isoelectric focusing using the NanoPro detection system also showed that GM6001 inhibited the LPA-induced phosphorylation of ERK1/2 in the E10 cells as shown in Figure 8B. In the SCC-9 cells, the MMP inhibitor did not affect phosphorylation of Akt, ERK or p38 (Figure 9A, middle), which is consistent with the lack of sensitivity to gefitinib in these cells (Figure 8A). In the D2 cells, like E10, GM6001 reduced LPA-induced phosphorylation of EGFR and Akt and completely blocked ERK phosphorylation (Figure 9A, right). We also examined the effect of the MMP inhibitor on migration. Treatment of the E10 cells with GM6001 strongly reduced LPA-induced cell migration (Figure 9C). In the SCC-9 cells, the pre-treatment gave only a partial and not significant reduction of the LPA-induced migration, corresponding to the lack of effect on Akt, ERK, and p38 phosphorylation. In D2 cells, GM6001 caused a small reduction of the basal, non-stimulated, cell migration (Figure 9C).

## Discussion

LPA can stimulate cell migration and invasion in several cancers, including ovarian, pancreatic, various gastrointestinal, and oral carcinomas [22,39]. Clarification of the mechanisms underlying LPA-induced cell migration in oral carcinomas is of considerable interest due to the fact that this cancer has a great tendency to spread by local invasion. In this study, we first sought to determine which receptors are involved in mediating the regulation of migration by LPA in two oral carcinoma cell lines. This is a complex issue, as six different LPARs have been identified [24] and relatively few selective tools for investigating the roles of the individual receptors in specific physiological and pathobiological processes are presently available. Furthermore, since we have found that in several cancer cells GPCR-conveyed mitogenic signals may be mediated by pathways involving PKC or EGFR, or both in interaction [20], we also investigated the roles of these mechanisms.

The present results suggested that, in the cell lines in which LPA stimulated migration, LPAR3 was involved in the effect. The E10 and the SCC-9 cells both expressed LPAR2 and 3, but no LPAR1 protein. The D2 cell line, which showed a slightly reduced migration upon LPA-stimulation, expressed both LPAR 1, 2 and 3 proteins. The LPAR1/3 inhibitor Ki16425 abolished the LPA-induced migration in both E10 and SCC-9 cells, suggesting that the LPAR3 receptor mediated the effect, given that no LPAR1 was detected. These results correlated well with inhibition of downstream signalling in these cells. When Ki16425 was developed and shown to have preference for LPAR1 and LPAR3, the only established LPA receptors were LPAR1-3, and therefore the effect of this inhibitor on other LPA receptors was not tested [40]. However, we are not aware of any later reports suggesting that Ki16425 acts on other receptors than LPAR1 or LPAR3. Ki16425 was also found to inhibit EGF-induced migration in the E10 and SCC-9 cells. This could indicate that the inhibitor was partially unspecific. However, in human ovarian carcinoma cells, Snider et al. showed that EGF induced LPA production, and the effect of EGF on migration was inhibited by Ki16425 [41]. Thus, if part of the EGF-induced migration is dependent on the secondary LPA production in our experiments as well, this would explain why inhibition of LPA receptors might reduce some of the EGF-mediated cell migration. The LPA agonist VPC31143(R) stimulated ERK phosphorylation and migration to about the same extent as LPA (Figure 3). This agonist was originally thought to be specific towards LPAR1 [35], but has more recently been shown to act via all the LPA receptors (LPAR1-6) like LPA itself [34]. Most important, however, we could also show that (2S)-OMPT, which has specificity for LPAR3 [42,43], stimulated ERK and Akt phosphorylation as well as migration in a manner similar to LPA (Figure 3). In contrast, the LPAR2-specific agonist LP-105 [44], did not mimic the effects of LPA (Figure 3). Taken together, these results suggest an involvement of LPAR3 in LPA-stimulated migration in E10 and SCC-9 oral carcinoma cells. However, the results could suggest that upon downregulation of LPAR3 with siRNA in the E10 cells, LPAR1 may substitute for LPAR3, but we have insufficient evidence for this.

We are not aware of other studies of receptors involved in LPA-induced migration in oral carcinoma cells. Studies in other cells have yielded varying results. LPAR3 has been implicated in ovarian cancer progression and cell migration [45,46], but was also reported to inhibit cell migration and invasion in colon cancer cells [47]. LPAR1 has been found to induce migration in cells from breast cancer [48], pancreatic cancer [49], and hepatocellular carcinoma [50] while it inhibited metastasis and invasion in prostate organotypic models [51]. LPAR2 was found to mediate LPA-induced invasion in endometrial cancer [52], but seemed to have an inhibitory role in pancreatic cancer [49]. In breast carcinoma cells both LPAR1 and 2 mediated LPA-induced migration, where LPAR1 worked at lower LPA-concentrations than LPAR2 and thus contributed to an effect over wider concentration ranges [53]. For the non-EDG LPA-receptors, LPAR4-6, information on their role in cancer is very limited and few studies exist. LPAR4 has shown both antimigratory [54,55] and proinvasive effects [56]. LPAR5 inhibited migration [29], and LPAR6 (synonymous to P2Y5) was thought to be pro-cancerous [57].

PKC may be involved in cancer progression [36]. LPAR3, which was found to be the most likely mediator of the migratory effect of LPA in the oral cancer cells studied here, is known to couple to G_q_ and phospholipase C β (PLC-β) and can thus convey activation of PKC. The LPA activity mediated by PKC was assessed in our study by use of an inhibitor of PKC, GF109203X. The inhibitor totally abolished the LPA-induced cell migration in SCC-9 cells. Furthermore TPA, a direct activator of PKC, mimicked the effect of LPA in these cells, providing further support for a role of PKC. A different mechanism was seen in the E10 cells, where both PKC activation and EGFR transactivation were necessary for a full migratory response to LPA. In these cells, TPA induced a partial migratory response, while both the EGF- and LPA-induced migration was inhibited by the PKC inhibitor.

In both E10 and D2 cells, which have very different migratory responses to LPA, EGFR was rapidly transactivated in response to LPA. This was associated with phosphorylation of Akt and ERK. In contrast, the SCC-9 cells, sharing the pro-migratory outcome of LPA stimulation with the E10 cells, showed no evidence of LPA-induced EGFR transactivation, but LPA induced EGFR-independent phosphorylation of ERK and Akt. These results were strengthened by the finding that inhibition of MMP by GM6001 closely mimicked the effects of gefitinib and cetuximab in E10, but did not affect SCC-9. Taken together, these results strongly suggest that LPA elicits rapid EGFR transactivation in E10 and D2, but not in SCC-9 cells. The lack of EGFR transactivation in SCC-9 cells is in conflict with findings in another study where transactivation in these cells was reported [27]. Because of these discrepant results, we tested our SCC-9 cells for authenticity. According to the genotyping, our cells were not altered after leaving the ATCC. However, the absence of any evidence of transactivation of EGFR by LPA in SCC-9 within the first few minutes does not rule out the possibility that transactivation might occur after longer exposure to LPA. EGFR activity via GPCR with a longer lag time has been described. This might have relevance to our results, since the LPA-induced migration in the SCC-9 cells was inhibited by gefitinib and cetuximab (Figure 7C) and to some extent also by GM6001 (Figure 8C). It is conceivable that a time-dependent EGFR ligand production in these cells could occur during the 48 h observation, thus explaining the sensitivity to inhibition by gefitinib and cetuximab despite no evidence of transactivation in the short-term experiments.

## Conclusion

While studies in various cancers indicate that different receptors may be involved in LPA-regulated cell migration, our present results strongly suggest that in the two oral cancer cell lines where LPA stimulated the migration, E10 and SCC-9, the effect was mediated by LPAR3. However, the cells differed with respect to downstream pathways. In the E10 cells, the stimulation via LPAR3 led to a concerted activation of PKC and transactivation of EGFR, both of which being required for full migratory response. In the SCC-9 cells, activation of PKC was crucial for LPA-induced migration, while there was no evidence of EGFR transactivation, although activation of EGFR upon longer culturing was not excluded. In a third oral carcinoma cell line, D2, LPA caused rapid EGFR transactivation, like in E10 cells, but D2 cells have a very high migratory activity in the absence of any stimulation, and LPA is rather slightly inhibitory. Thus, although the data show some common features in the mechanisms involved in the response to LPA in these oral carcinoma cell lines, they clearly demonstrate that there are important differences. Further studies are required both to get a better understanding of the degree of heterogeneity in oral carcinomas in terms of LPA signalling and to explore mechanisms that might provide therapeutic targets.

## Competing interests

The authors declare that they have no competing interests.

## Authors’ contributions

IJB conceived of the study, carried out the migration and invasion studies, drafted the manuscript and participated in the design of the study. IHT carried out immunoassays, participated in the design of the study and helped revise the manuscript. MA carried out immunoassays, participated in the design of the study and helped revise the manuscript. JØ carried out the qPCR and the NanoPro experiments and helped revising the manuscript. DLS participated in the design of the study and helped revise the manuscript. TC conceived of the study, participated in the design of the study and helped revise the manuscript. All authors read and approved of the final manuscript.

## Pre-publication history

The pre-publication history for this paper can be accessed here:

http://www.biomedcentral.com/1471-2407/14/432/prepub
